# Some Things Are Rarely Discussed in Public – on the Discourse of Corruption in Healthcare: Comment on "We Need to Talk About Corruption in Health Systems"

**DOI:** 10.15171/ijhpm.2019.51

**Published:** 2019-06-24

**Authors:** Peter Stiernstedt

**Affiliations:** School of Law and Criminology, University of West London, London, UK.

**Keywords:** Corruption, Anti-corruption, Governance, Healthcare

## Abstract

In an editorial titled "We Need to Talk About Corruption in Health Systems" the authors Hutchinson, Balabanova, and McKee hope to encourage a wider conversation about corruption in the health sector. Such conversations are difficult to hold for at least five reasons; it is hard to define corruption; corruption may allow some fragile health systems to subsist, shifting blame – are those involved in anti-corruption research colluding with corrupt officials; the legitimacy of studying corruption; and, that far too little is known about how to tackle corruption. This commentary explores those reasons and concludes that the authors make a strong case for a more open and directed discussion about corruption.

The title of this commentary is borrowed from the opening sentence of an editorial by the authors; Hutchinson, Balabanova, and McKee.^1^ In it the authors wish to address the health communities’ *dirty secret*^2^ with the intention to encourage a wider conversation within the health sector. The editorial opens broadly establishing the nature of some aspects of our lives and discourse that we wish to keep private. An example given is how sexual abuse traditionally remained secret, and how the #MeToo movement has not only empowered victims to speak out but has also progressed the overall discourse of a sensitive topic. They stipulate that talking about abuse of different kinds facilitate the process of addressing and changing things for the better.

The focus under consideration here is the “abuse of entrusted power for private gain” – to use the definition of corruption by Transparency International.^3^ Thus, by shedding light on why conversations about corruption in the health sector are difficult to hold, the authors strive to nurture the conversation within and among the many national and international stakeholders. This is a laudable ambition which receives complete concurrence also from this author, a scholar with similar interests as a corruption researcher. The lingering question is; who benefits from long, technical discussions over why something that should work in theory but does not work in practice?

Breaking the status quo would mean to question it, and in doing so talking openly about and problematizing corruption. To that end, corruption is established as a problem also in the health sector where the authors lean on the findings from the 2013 corruption barometer by Transparency International. Here it was reported that in 42 of 109 countries surveyed, over 50% citizens viewed their health systems as corrupt or very corrupt.^4^ A member of the general public with little or no insight in to previous research into the measurement of corruption might be astonished at such a result, or sceptical, particularly around the inherent difficulties with measuring corruption using perceptions based indices.^5^ The figures, albeit from 2013, are striking and might inspire a detailed read of the whole editorial. Despite methodological flaws, the survey by Transparency International achieved a significant raising of awareness of the issue in the sector. However, when an academic or professional with a vested interest in corruption research suggests the importance of the findings, warning bells go off – or at least they should. To what end are these figures used; are we talking about just raising awareness or is it something more, like design and implementation of anti-corruption measures or even policy-making?

The authors, who have worked as researchers on an international project on corruption, have witnessed how when describing achievements health ministers rarely discuss the role of corruption and the weaknesses of governance that often underlies it. To that end the authors have identified five reasons to why fruitful conversations on corruption are difficult to hold; it is hard to define corruption, corruption may allow some fragile health systems to keep going, blame shifting – are those involved in anti-corruption research colluding with corrupt officials, the legitimacy of studying corruption, and, that far too little is known about how to tackle corruption.

First, it is hard to define corruption. It is here that conversations on corruption can get stuck – or at least lose their full potential of achieving some meaningful change where definitions proliferate and contradict or are noticeable by their absence. As pointed out by the authors the United Nations Convention against Corruption does not even try to define its subject, instead, is merely lists a number of corrupt practices.^6^ Acknowledging the many interpretations of the concept of corruption, the editorial also contains a definition developed by the Cochrane Collaboration^7^ as “The abuse or complicity in abuse, of public or private position, power or authority to benefit oneself, a group, an organization or others close to oneself; where the benefits may be financial, material or non-material.” While there are no arguments for this particular definition being more viable than any of the other more established versions it does present something more tangible and add to the overall understanding of the phenomenon. In terms of nurturing an open discussion perhaps this is the most appropriate approach as corruption can take many forms, not all of which may be recognized as corruption by everyone. The transformation from definitions to effective anti-corruption instruments and policy is obviously another question.

Second, corruption may allow some fragile health systems to keep going, and if corruption is removed without addressing other potential weaknesses in the health system an equitable delivery may suffer. An unintended consequence of the eradication of corruption to support the most vulnerable (in need of a functioning and equitable health system) could thus end up hurting them even more. This idea of corruption fundamentally constructed as problem-solving is dealt with in-depth by Marquette and Peiffer^8^ in their examination of why anti-corruption initiatives fail. They recognise that corruption can in fact offer a way of dealing with socio-economic problems, particularly in weak institutional environments. The proposed solution is that anti-corruption interventions need to better understand the functions that corruption may serve, and find alternative ways to solve the problems that people face. This approach resonates well with the overall objective of nurturing an open discussion to increase mutual understanding.

Third, blame shifting, where it is easy for those involved in corruption to blame other, less powerful actors as corrupt and in doing so deflect attention from themselves. The question of how to conduct research on corruption is not easily answered. Research can present the individual with the unexpected moral dilemma as to whether or not immediately to act upon what is being discovered but thereby risk losing valuable access to information. This information could perhaps not benefit those already affected but, over a period of time lead to both better policy and effective legislation. Alternatively, the researcher also runs the risk of becoming the target and effectively blamed for negatively influencing the delicate power balances. This may involve not just a risk for career and reputation but also to life and limb.

Fourth, there is a concern over the legitimacy of studying corruption and the risk of diverting attention from other, possibly more important, issues. This fourth reason is supported by a 2007 article by a Turkish scholar^9^ claiming that corruption may be a manifestation of a neoliberal attack on the state. Neoliberal is here interpreted as a politicised definition of corruption, where efforts are concentrated on fighting against legally definable forms of corruption. It should be noted, however, that when looking at the work of this scholar one is struck by the author’s own opposition of the ideas encapsulated in neoliberalism. In itself this anti-neoliberal stance is not an argument for neglecting to question the legitimacy of corruption studies, but rather a call for a more nuanced picture. The authors of the editorial clearly evidence that the dismantling of the public health system in Anglo-western societies in the Reagan-Thatcher era noticeably failed in terms of preventing corruption in the health sector. Nevertheless, questioning the legitimacy of corruption studies should arguably not be confined to any one governance system. Instead and in line with the overall message of the editorial such studies actively encourage an open and broad discourse of corruption.

Fifth and finally, despite years of efforts to uphold good governance, far too little is known about how to tackle corruption. The authors cite the Cochrane report on interventions to reduce corruption in the health sector that found no studies exist which provide empirical evidence of successful strategies reducing corruption. Going beyond the health sector, this seems to be true also on a general level, as U4 research^10^ show that most anti-corruption initiatives fail. Regardless of sector the causes of failure and success however seem to be similar; failure occurs because of “design-reality gaps,” a mismatch between the expectations built into the design of the anti-corruption initiative as compared to on-the-ground realities. Conversely, success can be achieved by minimising or closing those gaps, but beyond that it is the politics of the situation that determines success. The intricacies of such politics are recognised by the authors, partly due to the reluctance to speak openly about corruption. The authors argue that even if an agreement is reached to address corruption there is still an issue of effectively triaging the problem. Priorities must be balanced between what is practically achievable and politically viable.

The authors deduce the importance of understanding the reasons behind why corrupt practices thrive in the health sector. Such understanding is created by constantly asking questions about who benefits and in what way, as this could shed light on the underlying causes. Further, and perhaps most importantly, the more that is understood about the causes in general the more is also understood about the extent to which they can be changed. From here it becomes possible to develop pragmatic solutions.^11^ It is suggested that the Sustainable Development Goals (SDGs), committed by the governments of the world to be achieved by 2030, could be leveraged to tackle corruption in the health sector.^12^ Hence, even if corruption may not yet be spoken about fully and openly in the halls of power at least it is shown that there is scope to incorporate the subject in the SDGs. There are arguably many stakeholders within countries and the international community when it comes to health-related SDGs. Those could be unified through having a more open debate about corruption and how research can help bridge any design-reality gap. Through the work of Zyglidopoulos et al^13^ the authors outline four broad paths of corruption research; individual, organisational, national and cultural. While the paths are not claimed to be presented in order of importance, perhaps they are or at least should be treated as. In doing so the authors not only make a strong case for an open discussion about corruption but also provide a direction for that discussion. A direction that would allow policy-makers, academic researchers and health sector professionals to discuss some things that are rarely considered in public.

## Ethical issues

Not applicable.

## Competing interests

Author declares that he has no competing interests.

## Author’s contribution

PS is the single author of the paper.
